# Health Care Interventions Delivered Over the Internet: How Systematic was the Review?

**DOI:** 10.2196/jmir.8.2.e11

**Published:** 2006-06-30

**Authors:** Evan Mayo-Wilson

I found the title and description of the recent review by Griffiths et al. [1] misleading. The authors describe their paper as "a systematic review".	However, the article fails to cite several published (and indexed) trials of internet-delivered therapy (e.g. [2]).  The search strategy and inclusion criteria were neither transparent nor replicable.  The authors note that they did not "set out to identify every published eHealth intervention paper" yet give no reason to believe that the sample obtained is	representative of the population of studies being reviewed.
